# The role of sialidases in the pathogenesis of bacterial vaginosis and their use as a promising pharmacological target in bacterial vaginosis

**DOI:** 10.3389/fcimb.2024.1367233

**Published:** 2024-03-01

**Authors:** Liuyan Chen, Jiayue Li, Bingbing Xiao

**Affiliations:** ^1^ Department of Obstetrics and Gynecology, Peking University First Hospital, Beijing, China; ^2^ School of Medicine, University College Dublin, Dublin, Ireland

**Keywords:** bacterial vaginosis, *Gardnerella vaginalis*, sialidase, vaginal dysbiosis, pathogenesis

## Abstract

Bacterial vaginosis (BV) is an infection of the genital tract characterized by disturbance of the normally *Lactobacilli-*dominated vaginal flora due to the overgrowth of *Gardnerella* and other anaerobic bacteria. *Gardnerella vaginalis*, an anaerobic pathogen and the major pathogen of BV, produces sialidases that cleave terminal sialic acid residues off of human glycans. By desialylation, sialidases not only alter the function of sialic acid-containing glycoconjugates but also play a vital role in the attachment, colonization and spread of many other vaginal pathogens. With known pathogenic effects, excellent performance of sialidase-based diagnostic tests, and promising therapeutic potentials of sialidase inhibitors, sialidases could be used as a biomarker of BV. This review explores the sources of sialidases and their role in vaginal dysbiosis, in aims to better understand their participation in the pathogenesis of BV and their value in the diagnosis and treatment of BV.

## 1 Introduction

Bacterial vaginosis (BV) is caused by a disturbance to the vaginal flora in which *Gardnerella* and other anaerobic bacteria replace the normal vaginal microbiota dominated by *lactobacilli* (Ravel et al., 2011). Lactic acid, H2O2, bacteriocins, and biosurfactants, which are antimicrobial and anti-inflammatory products produced by *lactobacilli*, decreases along with the health-promoting *lactobacilli*. The increased pH of the vagina creates advantages for the proliferation of facultative and obligate anaerobes, including *Gardnerella*, *Atopobium*, *Mobiluncus*, *Prevotella*, *Streptococcus*, *Ureaplasma*, *Megasphaera* etc (Amabebe and Anumba, 2018). Meanwhile, the concentrations of short chain fatty acids (SCFAs, such as acetate, malonate and succinate) and amines (such as putrescine, cadaverine, and tyramine) produced by the overgrown anaerobes increase in parallel with bacterial abundance and species biodiversity (Srinivasan et al., 2015; Vitali et al., 2015). A recent study found that a combination of vaginal microbiota metabolites representing BV increased basal and toll-like receptor (TLR) -induced production of TNF-α, demonstrating their immune regulatory effects (Delgado-Diaz et al., 2020).

As a major obstetrical and gynecological concern, BV is associated with many negative health outcomes, such as infertility (Ravel et al., 2021), preterm delivery (Honest et al., 2004; Cauci and Culhane, 2011; Manns-James, 2011), pelvic inflammatory disease (Taylor et al., 2013; Ravel et al., 2021), and sexually transmitted infections (Bautista et al., 2016; Armstrong and Kaul, 2021). Pathogenesis of BV involves degradation of the mucus layer on the surface of vaginal epithelium, exfoliation and detachment of the epithelial cells (Cauci et al., 2003), which in turn facilitates bacterial adhesion and biofilm formation (Swidsinski et al., 2005; Varki, 2009; Varki and Gagneux, 2012). Sialidases play a key role in the processes mentioned above, making sialidase activity measurement useful in the diagnosis and management of BV (Javed et al., 2019; Mabugana et al., 2023). Of course, mucus degradation is such a complex process that there are other glycosidases, proteases, and sulphatases involved in (Wiggins, 2001). For example, prolidase is a kind of proteolytic enzymes associated with BV, which shows a negative association with interleukin (IL)–8 levels in female CVF (Cauci et al., 2002) and can predict low birth weight and preterm birth with combination of vaginal pH and vaginal sialidase (Cauci et al., 2005).

As a major virulence factor of *Gardnerella* spp (Schellenberg et al., 2016; Kurukulasuriya et al., 2021), sialidases are important glycoside hydrolases that cleave sialic acid residues off of terminal glycans (Lewis et al., 2013; Robinson et al., 2019). Sialic acids are 9-carbon monosaccharides found in glycoconjugates such as glycoproteins and glycolipids, as well as at the distal end of *N-* and *O-*linked carbohydrate chains, also named glycans (Ghosh, 2020). As a part of glycoconjugates and substrates of sialidases, glycans have been found in human cervicovaginal fluid (CVF) (Moncla et al., 2015; Moncla et al., 2016; Wang et al., 2015) and surface of vaginal epithelial cells (Agarwal et al., 2023). Glycans heavily coat the surface of mammalian epithelial cells (Ochs et al., 2020; Argüeso et al., 2021), making them the frequent primary point of interaction between microorganisms and mucosal barriers (Poole et al., 2018). Through hydrolysis of sialic acids, which are highly electronegative carbohydrates, sialidases participate in many physiological and pathological pathways by lowering the surface charge of the whole cell, exposing glycoconjugates’ binding sites, changing the conformation of the glycoproteins, and eventually altering the functions of sialic acid-containing glycoconjugates (Pshezhetsky and Ashmarina, 2013).

Sialic acids support the defense barriers through a delicate balance between sialylation and desialylation (Cohen and Varki, 2010; Cao and Chen, 2012). Sialylation, mediated by sialyltransferases, is the addition of sialic acids to the end of oligosaccharides and glycoproteins, while desialylation, mediated by sialidases, is the removal of sialic acids. Sialoglycoproteins, composed of glycoproteins and sialic acids, are important defense components of the mucosal surface that create a physical barrier against pathogens (Lewis and Lewis, 2012). With a weight percentage of almost 16% sialic acids, mucins provide a dense physical barrier that disrupt the interactions between pathogens and epithelial cells (Slomiany et al., 1996; Moran et al., 2011). Moreover, sialylation also plays a role in immune response by altering the functions of immunoglobulins and regulating inflammation (Yoo and Morrison, 2005; Anthony and Ravetch, 2010).

Sialidases, also known as neuraminidases, have been detected in CVF and elevated level of sialidase activity is associated with BV (Briselden et al., 1992; Myziuk et al., 2003). In a 1992 study, women with BV had higher levels of sialidase activity in their vaginal secretions than those without (Briselden et al., 1992). Over the next three decades, many more studies produced similar results (Howe et al., 1999; Smayevsky et al., 2001; Cauci et al., 2003; Lewis et al., 2012). A recent study suggests that women with BV have higher sialic acid depletion and lower levels of sialylation (Agarwal et al., 2023), which could be explained by elevated sialidase activity as sialylation breaks down and depletes sialoglycans. Another study also detected roughly 3-fold lower amounts of total sialic acids and 3.5-fold greater amounts of free sialic acids in BV samples compared with normal samples using high-performance liquid chromatography (HPLC) (Lewis et al., 2012). However, the exact mechanism of sialidases causing BV is not fully understood, as the current understanding of the roles of vaginal epithelial glycans is still limited.

Besides BV, sialidases are involved in a broad spectrum of diseases within the human body as they can be produced by not only bacteria but also viruses, mammals, and protozoa. Bacterial sialidases also participate in host-bacteria interactions, coinfections, and dysbiosis in oral cavity, gastrointestinal tract and respiratory system (Siegel et al., 2014; Huang et al., 2015; Wong et al., 2018). Influenza A and B viruses can also produce sialidases, which in turn facilitates the development of influenza (Zambon, 2001). In mammals, sialidases are involved in a wide range of health issues, including cancers (SoÈnmez et al., 1999; Zhou et al., 2020), diabetes (Natori et al., 2013), neurodegenerative disorders (Liao et al., 2020; Khan et al., 2021), fibrosing diseases (Karhadkar et al., 2022) and heart diseases (Zhang et al., 2018; Chen et al., 2021).

As the catalytic activity of sialidases is essential to the colonization and dissemination of several pathogenic microorganisms, sialidases could be used as a promising diagnostic marker for BV (Briselden et al., 1992; Smayevsky et al., 2001). This article aims to review relevant literature to explore the characteristics of sialidases in CVF, their contributions to vaginal dysbiosis, and their clinical use in BV diagnosis and treatment.

## 2 Sources of sialidase activity

So far research has reported *in vitro* sialidase activity in some BV-associated bacteria (BVAB), such as isolates of *Prevotella*, *Bacteroides*, and *Gardnerella* (Briselden et al., 1992). Studies have illustrated the ability to produce sialidases by every strain of *Prevotella bivia*, while only some *G.vaginalis* isolates produce sialidases (Briselden et al., 1992; Lopes Dos Santos Santiago et al., 2011). However, *G.vaginalis* is able to produce higher levels of sialidases, demonstrated in a study of C57BL/6 mouse models where *Prevotella* models showed similar levels of sialidase activities with *G.vaginalis* in a 100 times infection titer compared to *Gardnerella*-colonized models (Gilbert et al., 2019). Apart from the abundance of bacteria themselves, other factors, such as sialidase expression levels, individual heterogeneity, and interactions between bacteria, might also affect sialidase activity in the CVF. Furthermore, sialidase produced by possible viruses and the host should be taken into consideration though there are few studies about this.

Among genotypes of *G.vaginalis*, the expression levels of sialidases are highly heterogeneous (Schellenberg et al., 2016). Based on quantitative polymerase chain reaction (qPCR) targeting clade-specific genes, *Gardnerella* is divided into four clades, clade 1 (encoding putative a-L-fucosidase), clade 2 (encoding a hypothetical protein), clade 3 (encoding thioredoxin) and clade 4 (encoding CIC family chloride transporter) (Balashov et al., 2014). They are different in sialidase activity: clade 2 have the highest activity followed by clade 1, clade3, and clade 4 (Qin and Xiao, 2022). In clade 4, the proposed sialidase encoding gene *sialidase A* gene is not detected (Shipitsyna et al., 2019).

Three sialidase homologs, NanH1 (also known as sialidase A), NanH2, and NanH3, have been identified in *G.vaginalis* (Janulaitiene et al., 2018; Robinson et al., 2019). Sialidase activity in *G.vaginalis* was initially thought to derive from sialidase encoding gene *nanH1* (Lopes Dos Santos Santiago et al., 2011), while a more recent study concludes that *nanH2* and *nanH3* are the primary sources of sialidase activity in *G.vaginalis* (Robinson et al., 2019). Schellenberg et al (Schellenberg et al., 2016). found that using a filter spot assay, the presence of *nanH1* was not indicator of sialidase activity: only 36 of the 77 *G.vaginalis* isolates that tested positive for *nanH1* actually produced sialidases. Meanwhile in another test done by polymerase chain reaction (PCR), sialidase activity in a collection of 34 isolated *G.vaginalis* strains was consistent with the detection of *nanH2* or *nanH3* (Robinson et al., 2019). The main functional distinction between NanH2 and NanH3 is that, NanH2 cleaves 9-O-acetylated sialic acid substrates far more efficiently than NanH3, either *in vitro* or *in vivo* (Robinson et al., 2019). In addition, *nanH3* is more commonly present than *nanH2* (Cauci et al., 2003). These results suggest that NanH2 and NanH3 are more likely to be the primary sources of sialidase activity in *G.vaginalis* in human CVF, whereas NanH1 contributes little.

Studies propose that the absence of sialidase activity by *nanH1* could be due to transcriptional regulation (Janulaitiene et al., 2018) and a lack of signal sequence, suggesting an intracellular localization of *nanH1* (Kurukulasuriya et al., 2021). However, limited evidence supports these hypotheses. Additionally, elevated *nanH1* gene levels have been found to be associated with both high-risk human papillomavirus (HPV) (Di Paola et al., 2017) and BV (Hardy et al., 2017). Thus, more research is needed to better understand the roles of the sialidase encoding genes besides sialidases expression.

## 3 Pathogenicity of sialidases

**Figure 1:**
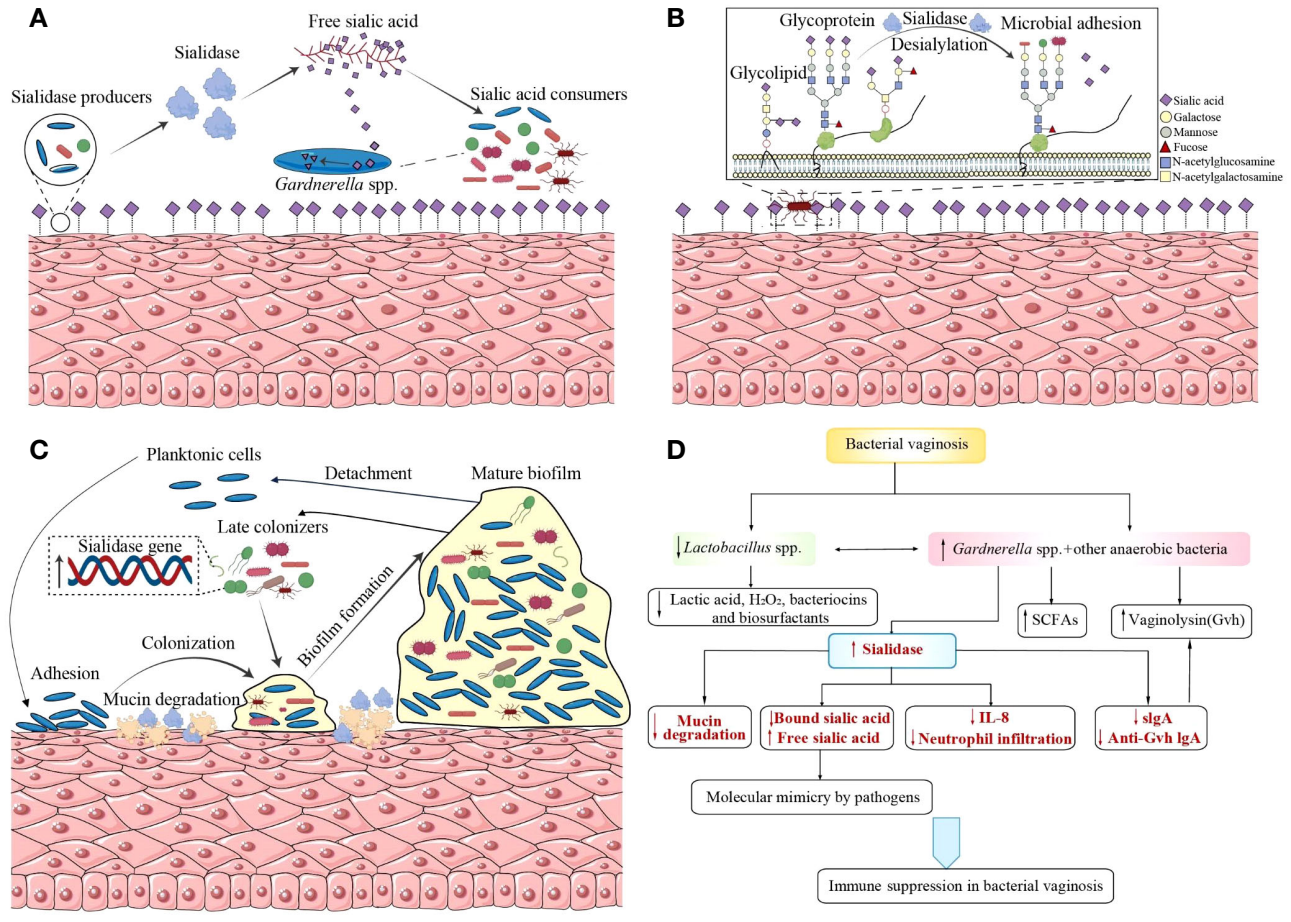
Sialidases’ participation in the pathogenesis of *Gardnerella vaginalis* and bacterial vaginosis. **(A)** Sialidase producers catalyze sialic acids from glycoconjugates as nutrition source for sialic acid consumers. **(B)** Desialylation of glycoconjugates by sialidases exposes new glycan epitopes for bacterial recognition and adhesion. **(C)** *G.vaginalis* and BVAB bacteria establish synergistic interactions based on sialidases during the formation of a polymicrobial biofilm. **(D)** Sialidases participate in the immune regulation of BV, supported by other hydrolytic enzymes, virulence, and immunomodulatory metabolites. BVAB, bacterial vaginosis-associated bacteria; SCFAs, short chain fatty acids; Gvh, *Gardnerella vaginalis* hemolysin; IL, interleukin; Anti-Gvh IgA, immunoglobulin A against *Gardnerella vaginalis* hemolysin.

The host mucosal defense barrier, which is important in the identification, integration, and elimination of pathogens, can be destroyed by desialylation of glycoconjugates such as mucins, cellular receptors, and immunoglobulins, which in turn facilitates bacterial adherence, colonization, invasion, and tissue breakdown (Briselden et al., 1992; Cauci et al., 2003, 1998; Cauci and Culhane, 2011). Sialidases’ participation in the pathogenesis of *G.vaginalis* and BV is discussed below (**Figure 1**).

### 3.1 Source of nutrition in bacteria

Bacteria can use free sialic acids, a hydrolysate of glycoconjugates catalyzed by sialidases, as a source of carbon for their nutrition and colonization (**Figure 1A**) (Lewis et al., 2013; Agarwal et al., 2020; Agarwal and Lewis, 2021). Evidence from mouse models shows that free sialic acids released by sialidases promote the growth of group B *Streptococcus* and the spread of ascending vaginal tract infections (Pezzicoli et al., 2012; Gilbert et al., 2013). Bacteria lacking sialidase encoding genes can also benefit from sialoglycan in the vagina via sialidase producers such as *G.vaginalis* (Agarwal et al., 2020). Some bacteria, such as *Fusobacterium nucleatum* (Haines-Menges et al., 2015; Agarwal et al., 2020) and group B *Streptococcus* (Pezzicoli et al., 2012), have sialic acid transport or catabolic pathways despite being sialidase-negative themselves. Moreover, *F.nucleatum* can reinforce sialidase activity produced by *G.vaginalis* in both *ex vivo* and *in vitro* coculture studies. *G.vaginalis* titers exhibit a dose-dependent increase with higher inocula of *F.nucleatum* or increasing proportions of its cell-free supernatant in an *in vitro* coculture system of *F.nucleatum* and *G.vaginalis*, in which *G.vaginalis* could not survive itself. This suggests that *F.nucleatum* may secrete factors to facilitate *G.vaginalis* growth. Additionally, in comparison to cocultures with *F.nucleatum*, monocultures of *G.vaginalis* needed at least a 20,000-fold greater inoculum to be viable after an overnight incubation (Agarwal et al., 2020). Therefore, *F.nucleatum* and *G.vaginalis* form a mutually beneficial relationship based on their glycan cross-feeding mode, which promotes their colonization and contributes to vaginal dysbiosis.

There have also been reports of the cross-feeding between commensal bacteria in the gut. For example, *Bifidobacterium breve* UCC2003, which contains a functional Nan cluster for sialic consumption, can use the sialic acid produced by *Bifidobacterium bifidum* PRL201048 (Egan et al., 2014). Similarly, in the oral cavity, *Streptococcus gordonii* employs sialic acids as their only carbon source (Byers et al., 1996). During the coinfection of influenza and *Streptococcus pneumoniae* in the respiratory tract, sialic acids produced by influenza accelerate bacterial replication *in vivo* and stimulate pneumococcal proliferation (Siegel et al., 2014).

### 3.2 Exposure of receptor binding sites

Sialidases can also promote infections by damaging the protective physical and biochemical barriers against pathogens through exposure of receptor binding sites for adhesins and toxins. In the oral cavity, adhesion of *S.gordonii* to oral epithelial cells is greatly increased by the presence of *Streptococcus oralis* in a sialidase-dependent manner through exposure of cryptic receptors binding sites (Beighton and Whiley, 1990; Wong et al., 2018).

Sialic acids are typically found at the terminal position of glycans. They can shield the underlying sugars (mostly galactose residues) from recognition, and then breakdown or adherence. Sialidases in the vagina may reveal glycan epitopes by the depletion of sialic acids and the exposure of underlying sugars to the surface (**Figure 1B**). In both *N*- and *O-*linked glycans, sialic acids cap Gal residues bound to N-acetylglucosamine (GlcNAc) or N-acetylgalactosamine (GalNAc), which is not accessible on the epithelial surface unless treated with exogenous sialidases or using cells from BV-positive specimens (Agarwal et al., 2023). Desialylation of glycoconjugates by sialidases causes loss of or reveal of new glycan epitopes, affecting microbe binding and host immunological recognition (Varki and Gagneux, 2012). Bacterial adhesion occurs when terminal sugars are exposed with the degradation of glycans, in which process carbohydrate-binding proteins like lectins, previously predicted in *Gardnerella*, serve as mediums (Bonnardel et al., 2021). According to genome screening, a greater repertory of carbohydrate-binding proteins is produced by vaginal bacterial species that are linked to infection and inflammation, which may allow them to bind a greater variety of glycans in the vagina. Compared with commensals like *Lactobacillus crispatus*, the mean number of lectins per strain is approximately 2-fold higher among those regarded as potential and confirmed pathogens (including *Lactobacillus iners*, *G.vaginalis*, *Prevotella*, group B *Streptococcus*, and *Escherichia coli*) (Bonnardel et al., 2021). With the deepening of research on the surface polysaccharide structure of the vagina and the bacterial carbohydrate-binding proteins, comprehensive insights into host–microbe interactions will be reached.

### 3.3 Biofilms formation

A biofilm is an organized community of microorganisms encased in a extracellular matrix made of proteins, polysaccharides, and nucleic acids, that attaches to a biological surface (Flemming et al., 2016; Jung et al., 2017) and contributes to the survival of bacterial infections (Del Pozo, 2018). Vaginal biofilms contribute to the persistence and recurrence of BV, as well as antibiotic resistance (Swidsinski et al., 2008; He et al., 2021). According to a recent research, 11 of the 24 *G.vaginalis* strains were able to form biofilms, providing themselves with advantages to evade host defense mechanisms and survive against antibiotics (Ma et al., 2022). An *in vitro* study suggests that most of the BVAB have a tendency to grow biofilms, and *G.vaginalis* has greater propensity to form a biofilm, enhancing its virulence potential through increased adhesion and cytotoxicity of epithelial cells compared to other anaerobes (Alves et al., 2014).

The lifecycle of biofilm formation is considered to include several stages: (i) adhesion to a surface, (ii) production of extracellular matrix, bacterial aggregation and biofilm accumulation until the development of a mature biofilm structure, and finally (iii) detachment (Joo and Otto, 2012). The initial adherence to vaginal epithelial cells has been acknowledged to be a necessary process to elicit BV (Swidsinski et al., 2005). As a dominant component of BV biofilm, *Gardnerella* spp. replaces pre-dominant *L.crispatus*, initiate bacterial colonization on vaginal epithelium and then serve as a scaffold for the attachment of other BVAB, including *Atopobium vaginae* (found in 80% of the samples and compromises 40% of the biofilm mass) and other heterogeneously mixed bacteria belonging to the *Bacteroides*, *Corynebacterium*, *Lactobacillus*, *Staphylococcus*, Streptococcus genera and so on (Castro et al., 2015; Castro et al., 2019; Swidsinski et al., 2005; Verstraelen and Swidsinski, 2013; Schwebke et al., 2014). The process is known as coaggregation (**Figure 1C**). Sialidases serve as a trigger at this initial stage of colonization. By means of its mucinase activity, the enzymes alter the characteristics of mucus discharges, catalyze them as a meal for bacteria and expose adhesion receptors on polysaccharides to promote bacterial colonization, increasing the potential for *G.vaginalis* to contact closely with the epithelium. Then early biofilm forms with the aggregation of other BVAB and the accumulation of extracellular matrix (Verstraelen and Swidsinski, 2013). Though *sialidase A* gene is not found to be associated with sialidase activity, it has been found to be associated with the presence of *G.vaginalis* biofilms, suggesting its possible contribution to biofilm formation (Hardy et al., 2017). There is still a lack of research comparing the expression levels of sialidase in biofilms and planktonic cells, which can provide us with deeper insights into the role of sialidase in biofilm formation. What’s more, interactions between the microorganisms within vaginal biofilms are worth investigating as sialidase activity may be affected by those sialidase-negative bacteria. Besides the finding that *F.nucleatum* and *G.vaginalis* benefit from each other, an *in vitro* dual-species biofilm model demonstrates that other BVAB, such as *Actinomyces neuii* and *Enterococcus faecalis*, can upregulate sialidase and vaginolysin expression in *G.vaginalis* to reinforce its virulence (Castro et al., 2019).

Similar findings of the involvements of sialidases in biofilm formation also presents in infections of other systems. In the early phases of pulmonary infection*, Pseudomonas aeruginosa* and its sialidases, existing on the highly sialylated surfaces of the upper respiratory tract, can target bacterial glycoconjugates, promote cell-cell interactions, and initiate biofilm formation (Soong, 2006). Viral sialidase inhibitors have demonstrated the ability to block the process of biofilm formation in clinical *in vitro*, suggesting a potential novel pharmacological target in bacterial pneumonia prevention (Soong, 2006). In *Porphyromonas gingivalis*, the main pathogenic bacterium in chronic periodontitis, the sialidase encoding gene shows a higher expression level than that in planktonic cells (Lo et al., 2009). Sialidase-deficient strains also demonstrates less and discontinuous biofilm formation compared with wild-type *P.gingivalis* strains (Xu et al., 2017).

### 3.4 Immune regulation

The host-mucosa-sialidase can be regarded as a whole because sialidase functions on the mucosa. Sialidase is central to the suppression and overwhelm of host immune response. Meanwhile, it is also supported by other hydrolytic enzymes, vaginolysin, and immunomodulatory metabolites (**Figure 1D**) (Amabebe and Anumba, 2022). Sialylation of glycoconjugates, such as mucins, immunoglobulins (especially secretory immunoglobulin A, sIgA), and cytokines, cleave the molecules’ terminal sialic acids and uncover their carbohydrate residues to all kinds of glycosidases, thus making them more susceptible to proteolytic degradation and hampering immune response against bacteria (Cauci et al., 2003; Cauci, 2004). For example, during the incubation of sIgA and BV vaginal specimens, the release of products with lower molecular weight into the extracellular environment are observed and the phenomenon can be reproduced by adding three exogenous enzymes: sialidase, *β*-galactosidase and hexosaminidase, which suggests the deglycosylation and proteolysis of sIgA in BV (Lewis et al., 2012).

In BV-positive women with a specific IgA immune response against *G.vaginalis* hemolysin (Gvh, vaginolysin), increased cleavage of IgA and a 5-fold higher sialidase activity is observed compared to those with a weaker IgA response (Cauci et al., 1998). Later, another study reconfirmed that elevated sialidase and prolidase levels reduce this mucosal adaptive immune response. Vaginolysin, another virulence factor of *G.vaginalis*, is a cholesterol-dependent cytolysin (CDC) which forms pores on cell membranes, free host intracellular contents and disrupts genital epithelial cells (Morrill et al., 2023). The immunosuppression allows vaginolysin to fully carry out its cytolytic action, which results in the detachment and destruction of the vaginal epithelial cells that eventually produce clue cells (Castro et al., 2019).

High sialidases and prolidases levels are also associated with elevated vaginal IL-1β, leading to tissue damage and increased susceptibility to sexually transmitted infections (STIs) (Cauci et al., 2008). Despite that IL-1β stimulates IL-8 secretion, sialidase level is also inversely correlated to vaginal IL-8 and neutrophils, which inhibits neutrophil infiltration and the proinflammatory cascade (Cauci et al., 2008). According to *in vivo* research, BVAB can evade the immune response by either secreting molecules that aid in the breakdown of IL-8 or by suppressing the generation and stability of IL-8 (Santos et al., 2018). These findings suggest that in BV-positive women, sialidases contribute to the suppression of innate mucosal immunity.

However, BVAB induced the secretion of IL-6, IL-8, G-CSF, IP-10, MIP-1β, RANTES, and Gro-α, while *lactobacilli* did not in another study that used a coculture model to characterize the response of vaginal epithelial cells to a series of vaginal bacteria, including commensal *lactobacilli* and BVAB such as *G.vaginalis*, *A.vaginae*, *Mobiluncis curtisii*, and *P.bivia* (Eade et al., 2012). The results is consistent with that *A.vaginae* induces a robust proinflammatory response by elevating transcript levels of IL-6, IL-8, and antimicrobial peptide β-defensin 4 (Libby et al., 2008). It seems that BVAB trigger mucosal innate immune response, increasing production of cytokines and defensins to eliminate pathogens. But excessive inflammatory response might lead to a disturbance of the vaginal immunological barrier and increasing susceptibility to STIs (Doerflinger et al., 2014).

Furthermore, bacterial surface sialylation may serve as an immunological mask (Ram et al., 2017; Vimr and Lichtensteiger, 2002). It has been proposed that bacteria might be passed for host cells and evade the host’s immune system by incorporating the cleaved sialic acids into their cell surface structures (Varki and Gagneux, 2012). Differentiation between self-sialic-acids and close mimics is achieved through intrinsic lectins such as sialic acid-binding immunoglobulin-like lectins (Siglecs) anchored on most immune cells (Duan and Paulson, 2020). By engaging inhibitory Siglec‐5 and Siglec‐9, group B *Streptococcus* can escape from host immune responses (Carlin et al., 2007). *Neisseria gonorrhoeae* transfers sialic acid residues to its surface lipooligosaccharide (LOS) to achieve molecular simulation, which contributes to its serum resistance and complement resistance in all three pathways (classical, lectin, and alternative) (Ram et al., 2017). A study reports that vaginolysin is able to release the contents of cervical epithelial cells, promote gonococcal LOS acquisition of sialic acids, and evade complement attack through increased binding of the regulatory protein factor H (Morrill et al., 2023), suggesting that sialidases and vaginolysin are both crucial in the regulation of the LOS sialylation level and its pathogenic ability. Meanwhile, another study reports that desialylation of gonococcal LOS by sialidases in women promotes increased transmission of infection to men (Ketterer et al., 2016). These findings suggest that sialylation and desialylation may to have unique functions during the invasion of pathogens.

## 4 Sialidase and bacterial vaginosis

### 4.1 Sialidase and characteristics of BV

Elevated sialidase activity has been observed in BV CVF, suggesting that sialidases could be used as a promising biomarker for BV (Briselden et al., 1992). The presence of *sialidase A* gene was detected in all 24 *G.vaginalis* samples in a recent study (Ma et al., 2022), while another study reports an association between sialidase activity in molecular-BV (community state type IV, CST IV) and changes in the bacterial components of the local microbiome, assessed by using V3–V4 16S rRNA sequencing (Ferreira et al., 2022). *Gardnerella*, *Atopobium*, and *Prevotella* were among BV-associated the genera that were more prevalent in women with high sialidase activity (Ng et al., 2021). Increased sialidase may be attributed to the higher abundance of some BVAB that can produce sialidases by themselves, such as *Prevotella* (Briselden et al., 1992). At the same time, sialidases can impair the vaginal mucosal immune system, which creates a beneficial environment for the overgrowth of BVAB over the *Lactobacillus* spp. and increases bacterial diversity (Lewis et al., 2013).

### 4.2 Sialidase and diagnosis of BV

As a biomarker for BV, sialidases could be used to develop new diagnostic tests as cheaper and quicker alternatives to the current standard clinical diagnostic tools. Current clinical diagnosis of BV is often based on the Nugent scoring system (Nugent et al., 1991) or the Amsel criteria (Amsel et al., 1983), both of which require microscopy and trained professionals. On the contrary, enzyme-based simple assays may be cheaper and quicker (Robinson et al., 2019; Wu et al., 2019; Cortés-Sarabia et al., 2020; Rodríguez-Nava et al., 2021; Avila-Huerta et al., 2023; Liu et al., 2023). Several new tests have been developed to detect sialidases. A comparison of their clinical diagnostic performance is shown in **Table 1**. The most widely used is BVBlue test, a microscopy-independent bedside test that detects sialidase activity using ≥7.8 U as the cut-off value for diagnosis of BV (Myziuk et al., 2003; Bradshaw et al., 2005; Permsak et al., 2005; Madhivanan et al., 2014; Foessleitner et al., 2021). *OSOM^®^ BVBLUE^®^ Test* is a commercial chromogenic test that can rapidly detect elevated vaginal fluid sialidase activity, with excellent sensitivity and specificity compared to Gram Stain, and it is widely used in many parts of the world. Similarly, a sensitive colorimetric bioactive paper that changes its color from white to dark purple in the presence of sialidases demonstrates a quick reaction time and strong storage stability (Zhang and Rochefort, 2013), though its clinical performance in BV diagnosis was not evaluated. Although sialidase activity tests are performed clinically, the results are currently only used as references and not as a diagnostic criterion.

**Table 1:** Sialidase-based tests for BV and their clinical diagnostic performance.

Methods	Technique	Diagnostic Criteria	References	Sample Size	Sensitivity	Specificity	Positive Predictive Value	Negative Predictive Value
BVBlue test (Myziuk et al., 2003)	Chromogenic test	Sialidase activity≥7.8 U	the Nugent scoring	57	91.7%	97.8%	91.7%	97.8%
			the Amsel criteria		50.0%	100%	100%	88.2%
BVBlue test (Bradshaw et al., 2005)			the Nugent scoring	288	88%	95%	/	/
			the Amsel criteria		88%	91%	/	/
BVBlue test (Permsak et al., 2005)			the Nugent scoring	173	94%	96%	86%	98%
BVBlue test (Madhivanan et al., 2014)			the Nugent scoring	266	38%	95%	90%	54%
			the Amsel criteria	323	51%	94%	82%	78%
BVBlue test (Foessleitner et al., 2021)			the Nugent scoring	200	81%	100%	100%	98.1%
A sensitive colorimetric bioactive paper (Zhang and Rochefort, 2013)	Colorimetric biosensor	The changes of paper color from white to dark purple	/	/	/	/	/	/
PCRs of nanH2 or nanH3 (Robinson et al., 2019)	PCR	The dictation of nanH2 or nanH3 gene	the Nugent scoring	67	80.95%	78.26%	/	/
A turn-on tetravalent sialic acid-coated tetraphenylethene luminogen (TPE4S) (Liu et al., 2018)	Fluorescence response	Based on the relative fluorescence intensities (I/I_0_) monitored at 510 nm of experimental groups (I) and control group (I_0_) added 20 μM TPE4S, the samples are graded as normal (grade 1, 0< I/I_0_ ≤ 5), sialidase weak positive (grade 2, 5< I/I_0_ ≤ 10), and sialidase strong positive (grade 3, I/I_0_ > 10).	BVBlue test	150	92.5%	91.8%	/	/
A biochemiluminescent sialidase assay (Agarwal et al., 2020)	Biochemiluminescence	A cutoff value of 400,000 relative light units when a Helios 2000 luminometer is used.	the Amsel criteria	423	95.40%	94.94%	83%	98.76%
Boron and nitrogen codoped fluorescent carbon dots (BN-CDs) (Liu et al., 2023)	Fluorescence spectrometry	Sialidase concentration>1.25 U/mL	the Amsel criteria	6	/	/	/	/
Nanophotonic sialidase immunoassay (Rodríguez-Nava et al., 2021)	Immunosensing	Sialidase concentration>25.194 ng/mL	the Amsel criteria	162	96.29%	96.29%	92.86%	98.11%
A novel microfluidic paper-based analytical device (Avila-Huerta et al., 2023)	Immunosensing	Sialidase concentration>25.1ng/mL	the Nugent scoring&the Amsel criteria	14	/	/	/	/

/: the data was not provided.

#### 4.2.1 PCR

The *nanH3* gene expression could be used for PCR detection of BV as its level differs in normal microbiota and BV cervicovaginal fluid samples (Novak et al., 2023). PCR detection of *nanH2* or *nanH3* has a sensitivity of 80.95% and a specificity of 78.26% in differentiating between *Lactobacillus*-dominance and BV, as determined by Nugent scoring (Robinson et al., 2019). However, the test only detects sialidase produced by *G.vaginalis*, limiting its applicability to other BV pathogens.

#### 4.2.2 Fluorescence

Fluorescence could also be used to visualize sialic acids on cell membranes. The first test developed and adopted for BV diagnosis was turn-on tetravalent sialic acid-coated tetraphenylethene luminogen (Liu et al., 2018). Later on, a biochemiluminescent sialidase assay using a firefly luciferin derived substrate was developed, in which luciferins released by cleavage of the substrate subsequently oxidize and generate a light signal indicating relative sialidase concentration (Wu et al., 2019). More recently, a novel boron and nitrogen codoped fluorescent carbon dots (BN-CDs) was developed based on fluorescence spectrometry, in which sialidases can restore the fluorescence by interfering with the selective recognition interaction between the sialic acid and phenylboronic acid groups on the surface of BN-CDs, limiting fluorescence emission (Liu et al., 2023). The probe is comparable to Amsel criteria in its diagnosis of BV, indicating promising use for clinical diagnosis and therapy (Liu et al., 2023).

#### 4.2.3 Immunosensing

A new microfluidic paper-based analytical tool based on a monoclonal antibody that has a high specificity for sialidase recognition for BV diagnosis was described (Avila-Huerta et al., 2023). Taking advantage of a surface coated with graphene oxide as a fluorescence quencher, they developed a Y-shaped strip, consisting of an entrance, a control, and a test zone (Avila-Huerta et al., 2023). The apparatus can achieve a prompt and sensitive response within 20 minutes for the identification of BV, making it economically accessible and convenient for large scale use (Avila-Huerta et al., 2023).

Another research team recently designed and manufactured a monoclonal antibody (mAb) targeted against *G.vaginalis* sialidases (Cortés-Sarabia et al., 2020). They further developed a single-step quantitative biosensing system for BV diagnosis, using graphene oxide-coated microwells and mAb-decorated quantum dots (Rodríguez-Nava et al., 2021). Sialidase activity in vaginal swab samples detected by this method has a 96.29% specificity and 96.29% sensitivity, using Amsel criteria for the identification of BV (Rodríguez-Nava et al., 2021).

### 4.3 Sialidase and treatment of BV

With the understanding of the molecular mechanism of sialidases and its association with the pathogenesis of BV, sialidases can be used as not only a promising diagnostic marker but also a pharmaceutical target through activity blockage using inhibitors (Keil et al., 2022). Sialidase inhibitors include transition-state analogue inhibitors, mechanism-based inhibitors, suicide substrate inhibitors, product analogue inhibitors, and natural product inhibitors (Keil et al., 2022), which can act on virus, bacteria, human and protozoa sialidases. Numerous natural compounds have been identified and examined for their ability to inhibit sialidases from human, bacteria and influenza viruses. As for bacteria sialidases, three novel compounds as potent inhibitors are isolated from *Lespedeza bicolor* and effect in a dose-dependent manner, among them the best inhibitor has an IC_50_ (represents the compound concentration that causes 50% enzyme activity loss) of 0.09 μM (Woo et al., 2011). A recent discovery is a curcumin analogue against *S.pneumoniae* Nan A, whose IC_50_ value is 0.2 ± 0.1 μM, exhibiting a 3-fold increase in inhibitory efficacy compared to curcumin (Kim et al., 2018). Natural products provide us with an alternate source for creating novel bacterial sialidases inhibitors and treating sepsis caused by bacteria infections, which are worth exploring for BV treatment. In our discussion of potential treatment options for BV, with *G.vaginalis* being the major pathogen, we will be focusing on bacterial sialidase inhibitors (Keil et al., 2022) and salic acid analogs (Agarwal and Lewis, 2021).

#### 4.3.1 Sialidase inhibitors

Among them the most studied is the influenza virus sialidase inhibitors. Influenza sialidase (usually called neuraminidase) is required for the infection cycle to continue because it releases the freshly generated virus from the host cell, contributing to its spreading and preventing self-aggregation of the viral particles (Glanz et al., 2018). Currently, there are three antiviral drugs that target the glycoprotein neuraminidase on the surface of the influenza virus, including oseltamivir, zanamivir, and peramivir. They are essentially transition-state analogue inhibitors and work by inhibiting the neuraminidase enzyme’s activity and preventing the virus from exiting the infected cells (Javanian et al., 2021).

As bacterial and viral sialidases share the same sialic acid interaction sites, the ASP boxes (Roggentin et al., 1989), influenza virus sialidase inhibitors can be used to block the bacterial sialidase active site and prevent the formation of biofilms (Hayden et al., 1999). Evidence shows that influenza virus sialidase inhibitors oseltamivir and peramivir can block *P.aeruginosa* biofilm formation in a dose-dependent manner (Soong, 2006). Similarly, the desialylation of sIgA during incubations with BV samples and can be inhibited by deoxy-dehydro-sialic acid (DDSia), a synthetic sialidase inhibitor (Lewis et al., 2012). Zanamivir impairs the virulence of the BV-associated pathogen *G.vaginalis* through a reduction of 30% in *G.vaginalis* sialidase activity and 50% in its ability to invade host cells (Govinden et al., 2018). It’s interesting that the medicine for influenza treatment associates with BV. Anyway, they provide us with a new prospective to treat BV, despite neuraminidase inhibitor sensitivity varies throughout mammalian, microbial, and viral neuraminidases.

#### 4.3.2 Sialic acid analogs

**Figure 2:**
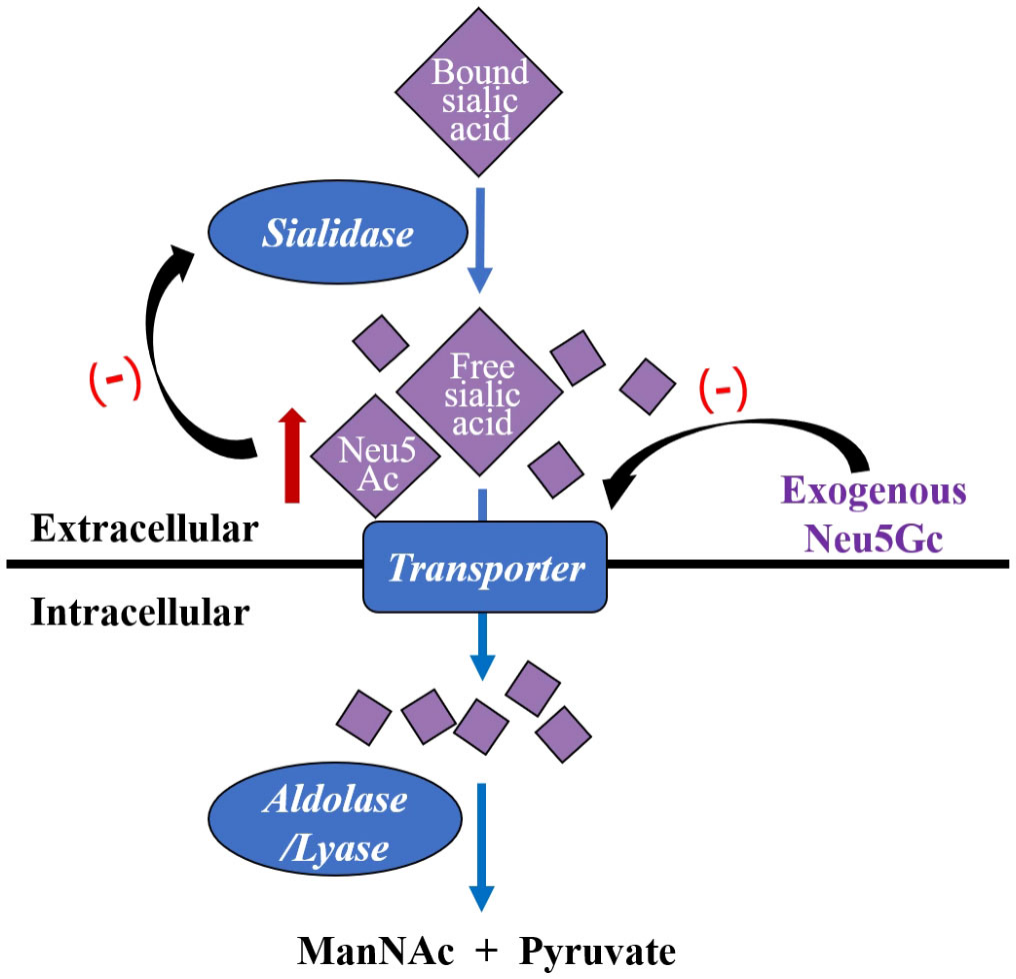
*Gardnerella vaginalis* captures free Neu5Ac hydrolyzed by sialidases, pumps them into the cell by a transporter, and then catalyze them into ManNac and pyruvate by intracellular aldolase/lyase. Exogenous Neu5Gc is a kind of sialic acid analogues, which inhibits *G.vaginalis* transporter and results in extracellular Neu5Ac accumulation. Neu5Ac is a weaker inhibitor of sialidases base on feedback mechanism. Neu5Ac, N-acetylneuraminic acid; Neu5Gc, N-glycolylneuraminic acid; ManNac, *N*-acetylmannosamine.

The two major forms of sialic acid in mammals, N-acetylneuraminic acid (Neu5Ac) and N-glycolylneuraminic acid (Neu5Gc), differ by a single oxygen atom, with Neu5Ac being the most prevalent form of sialic acid in mammalian cells (Schauer and Kamerling, 2018). The enzyme needed to synthesize Neu5Gc from Neu5Ac is called CMP-N-acetylneuraminic acid (CMP-NeuAc) hydroxylase, which is inactive in human, so Neu5Gc is a non-human derived sialic acid (Lewis et al., 2013). Once Neu5Ac is released by sialidases in the vagina, transport, uptaking and catabolism of them are proceeded within cells. The intracellular process is mediated by Neu5Ac lyase/aldolase and the substrates are catalyzed into N-acetylmannosamine (ManNAc) and pyruvate without accumulation (Lewis et al., 2013). An inherent biological mechanism for regulating enzyme processes is feedback inhibition through end-product inhibition of upstream enzymes. Through feedback inhibition, free Neu5Ac is a weak inhibitor of sialidases (Schauer and Kamerling, 1997). While a high-affinity transport mechanism in *G.vaginalis* has a preference for Neu5Ac, *G.vaginalis* sialidase does not appear to have strong preferences between Neu5Ac and Neu5Gc as substrates (Byers et al., 1999). This means that the uptake and breakdown of sialic acids are substrate-dependent and occur far more slowly and incompletely, when there is a substantial concentration of Neu5Gc (**Figure 2**). Later a study confirmed that *G.vaginalis* could liberate free Neu5Ac from IgA but fails to consume them with the presence of Neu5Gc, which further indicates Neu5Gc’s potential as an inhibitor to reduce Neu5Ac transport into *G.vaginalis* (Lewis et al., 2013). These findings are consistent with a prior discovery that in the bacterium *S.oralis*, Neu5Gc inhibits the uptake of Neu5Ac (Byers et al., 1999). Despite that Neu5Gc shows sialidase inhibitory activity, its effects for anti-BV are not verified and its effectiveness and safety still need experimental verification.

## 5 Conclusion

This study explores the role of sialidases in vaginal dysbiosis, pathogenesis of BV, and promising diagnostic and treatment options for BV. Although the composition and dynamics of the human vaginal microbiome are being studied more and more, we still know little about the mechanisms underlying the development of vaginal dysbiosis and the critical factors that influence it. As a main virulence factor of *Gardnerella* spp. and an important glycoside hydrolase enzyme, sialidases cleave sialic acid from terminal glycans, also known as desialylation. The process facilitates the destruction of mucosal defense barrier, as well as bacterial adhesion, colonization, and invasion into the vaginal epithelia through provision of nutrient sources, exposure of receptor binding sites, biofilms formation, and immunity regulation. However, not all *G.vaginalis* strains can produce sialidases and the contribution of sialidases to BV is just part of the pathogenesis of *G.vaginalis*. There are still other BVAB, virus, and even the human body itself can produce sialidases. Moreover, the use of sialidases as a biomarker for predicting treatment outcomes and the prognosis of BV still needs to be tested in clinical studies. Future research should focus on understanding the pathogenesis of sialidases produced by different strains of *G.vaginalis* and other sources, as well as the association between sialidases and the persistence and recurrence of BV, to provide new insights to improve diagnosis and treatment of BV.

## Author contributions

LC: Writing – original draft, Writing – review & editing. JL: Writing – review & editing. BX: Conceptualization, Writing – review & editing, Funding acquisition.

## References

[B1] Agarwal K. Choudhury B. Robinson L. S. Morrill S. R. Bouchibiti Y. Chilin-Fuentes D.. (2023). Resident microbes shape the vaginal epithelial glycan landscape. Sci. Trans. Med. 15, eabp9599. doi: 10.1126/scitranslmed.abp9599 PMC11419735 38019934

[B2] Agarwal K. Lewis A. L. (2021). Vaginal sialoglycan foraging by *Gardnerella vaginalis* : mucus barriers as a meal for unwelcome guests? Glycobiology 31, 667–680. doi: 10.1093/glycob/cwab024 33825850 PMC8252861

[B3] Agarwal K. Robinson L. S. Aggarwal S. Foster L. R. Hernandez-Leyva A. Lin H.. (2020). Glycan cross-feeding supports mutualism between Fusobacterium and the vaginal microbiota. PloS Biol. 18, e3000788. doi: 10.1371/journal.pbio.3000788 32841232 PMC7447053

[B4] Alves P. Castro J. Sousa C. Cereija T. B. Cerca N. (2014). Gardnerella vaginalis outcompetes 29 other bacterial species isolated from patients with bacterial vaginosis, using in an *in vitro* biofilm formation model. J. Infect. Dis. 210, 593–596. doi: 10.1093/infdis/jiu131 24596283

[B5] Amabebe E. Anumba D. O. C. (2018). The vaginal microenvironment: the physiologic role of lactobacilli. Front. Med. 5. doi: 10.3389/fmed.2018.00181 PMC6008313 29951482

[B6] Amabebe E. Anumba D. O. C. (2022). Mechanistic insights into immune suppression and evasion in bacterial vaginosis. Curr. Microbiol. 79, 84. doi: 10.1007/s00284-022-02771-2 35128579 PMC8818625

[B7] Amsel R. Totten P. A. Spiegel C. A. Chen K. C. S. Eschenbach D. Holmes K. K. (1983). Nonspecific vaginitis. Am. J. Med. 74, 14–22. doi: 10.1016/0002-9343(83)91112-9 6600371

[B8] Anthony R. M. Ravetch J. V. (2010). A novel role for the igG fc glycan: the anti-inflammatory activity of sialylated igG fcs. J. Clin. Immunol. 30, 9–14. doi: 10.1007/s10875-010-9405-6 20480216

[B9] Argüeso P. Woodward A. M. AbuSamra D. B. (2021). The epithelial cell glycocalyx in ocular surface infection. Front. Immunol. 12. doi: 10.3389/fimmu.2021.729260 PMC8419333 34497615

[B10] Armstrong E. Kaul R. (2021). Beyond bacterial vaginosis: vaginal lactobacilli and HIV risk. Microbiome 9, 239. doi: 10.1186/s40168-021-01183-x 34893070 PMC8665571

[B11] Avila-Huerta M. D. Leyva-Hidalgo K. Cortés-Sarabia K. Estrada-Moreno A. K. Vences-Velázquez A. Morales-Narváez E. (2023). Disposable device for bacterial vaginosis detection. ACS Meas. Sci. Au 3, 355–360. doi: 10.1021/acsmeasuresciau.3c00007 37868361 PMC10588930

[B12] Balashov S. V. Mordechai E. Adelson M. E. Gygax S. E. (2014). Identification, quantification and subtyping of Gardnerella vaginalis in noncultured clinical vaginal samples by quantitative PCR. J. Med. Microbiol. 63, 162–175. doi: 10.1099/jmm.0.066407-0 24200640

[B13] Bautista C. T. Wurapa E. Sateren W. B. Morris S. Hollingsworth B. Sanchez J. L. (2016). Bacterial vaginosis: a synthesis of the literature on etiology, prevalence, risk factors, and relationship with chlamydia and gonorrhea infections. Military Med. Res. 3, 4. doi: 10.1186/s40779-016-0074-5 PMC4752809 26877884

[B14] Beighton D. Whiley R. A. (1990). Sialidase activity of the “Streptococcus milleri group” and other viridans group streptococci. J. Clin. Microbiol. 28, 1431–1433. doi: 10.1128/jcm.28.6.1431-1433.1990 2199505 PMC267946

[B15] Bonnardel F. Haslam S. M. Dell A. Feizi T. Liu Y. Tajadura-Ortega V.. (2021). Proteome-wide prediction of bacterial carbohydrate-binding proteins as a tool for understanding commensal and pathogen colonisation of the vaginal microbiome. NPJ Biofilms Microbiomes 7, 49. doi: 10.1038/s41522-021-00220-9 34131152 PMC8206207

[B16] Bradshaw C. S. Morton A. N. Garland S. M. Horvath L. B. Kuzevska I. Fairley C. K. (2005). Evaluation of a point-of-care test, BVBlue, and clinical and laboratory criteria for diagnosis of bacterial vaginosis. J. Clin. Microbiol. 43, 1304–1308. doi: 10.1128/JCM.43.3.1304-1308.2005 15750100 PMC1081297

[B17] Briselden A. M. Moncla B. J. Stevens C. E. Hillier S. L. (1992). Sialidases (neuraminidases) in bacterial vaginosis and bacterial vaginosis-associated microflora. J. Clin. Microbiol. 30, 663–666. doi: 10.1128/jcm.30.3.663-666.1992 1551983 PMC265128

[B18] Byers H. L. Homer K. A. Beighton D. (1996). Utilization of sialic acid by viridans streptococci. J. Dent. Res. 75, 1564–1571. doi: 10.1177/00220345960750080701 8906124

[B19] Byers H. L. Homer K. A. Tarelli E. Beighton D. (1999). N-Acetylneuraminic acid transport by Streptococcus oralis strain AR3. J. Med. Microbiol. 48, 375–381. doi: 10.1099/00222615-48-4-375 10509480

[B20] Cao H. Chen X. (2012). “General consideration on sialic acid chemistry,” in Carbohydrate microarrays, methods in molecular biology. Ed. Chevolot Y. (Humana Press, Totowa, NJ), 31–56. doi: 10.1007/978-1-61779-373-8_3 PMC11288308 22057516

[B21] Carlin A. F. Lewis A. L. Varki A. Nizet V. (2007). Group B streptococcal capsular sialic acids interact with siglecs (Immunoglobulin-like lectins) on human leukocytes. J. Bacteriol 189, 1231–1237. doi: 10.1128/JB.01155-06 16997964 PMC1797352

[B22] Castro J. Alves P. Sousa C. Cereija T. França Â. Jefferson K. K.. (2015). Using an *in-vitro* biofilm model to assess the virulence potential of Bacterial Vaginosis or non-Bacterial Vaginosis Gardnerella vaginalis isolates. Sci. Rep. 5, 11640. doi: 10.1038/srep11640 26113465 PMC4481526

[B23] Castro J. MaChado D. Cerca N. (2019). Unveiling the role of Gardnerella vaginalis in polymicrobial Bacterial Vaginosis biofilms: the impact of other vaginal pathogens living as neighbors. ISME J. 13, 1306–1317. doi: 10.1038/s41396-018-0337-0 30670827 PMC6474217

[B24] Cauci S. (2004). Vaginal immunity in bacterial vaginosis. Curr. Infect. Dis. Rep. 6, 450–456. doi: 10.1007/s11908-004-0064-8 15538982

[B25] Cauci S. Culhane J. F. (2011). High sialidase levels increase preterm birth risk among women who are bacterial vaginosis–positive in early gestation. Am. J. Obstet. Gynecol. 204, 142.e1–142.e9. doi: 10.1016/j.ajog.2010.08.061 21055720

[B26] Cauci S. Culhane J. F. Di Santolo M. McCollum K. (2008). Among pregnant women with bacterial vaginosis, the hydrolytic enzymes sialidase and prolidase are positively associated with interleukin-1β. Am. J. Obstet. Gynecol. 198, 132.e1–132.e7. doi: 10.1016/j.ajog.2007.05.035 17714681

[B27] Cauci S. Driussi S. Monte R. Lanzafame P. Quadrifoglio F. (1998). Immunoglobulin A response against Gardnerella vaginalis hemolysin and sialidase activity in bacterial vaginosis. Am. J. Obstet. Gynecol. 178, 511–515. doi: 10.1016/S0002-9378(98)70430-2 9539518

[B28] Cauci S. Guaschino S. Driussi S. De Santo D. Lanzafame P. Quadrifoglio F. (2002). Correlation of Local Interleukin-8 with Immunoglobulin A against *Gardnerella vaginalis* Hemolysin and with Prolidase and Sialidase Levels in Women with Bacterial Vaginosis. J. Infect. Dis. 185, 1614–1620. doi: 10.1086/340417 12023767

[B29] Cauci S. McGregor J. Thorsen P. Grove J. Guaschino S. (2005). Combination of vaginal pH with vaginal sialidase and prolidase activities for prediction of low birth weight and preterm birth. Am. J. Obstet. Gynecol. 192, 489–496. doi: 10.1016/j.ajog.2004.07.023 15695992

[B30] Cauci S. Thorsen P. Schendel D. E. Bremmelgaard A. Quadrifoglio F. Guaschino S. (2003). Determination of Immunoglobulin A against *Gardnerella vaginalis* Hemolysin, Sialidase, and Prolidase Activities in Vaginal Fluid: Implications for Adverse Pregnancy Outcomes. J. Clin. Microbiol. 41, 435–438. doi: 10.1128/JCM.41.1.435-438.2003 12517887 PMC149625

[B31] Chen Q.-Q. Ma G. Liu J.-F. Cai Y.-Y. Zhang J.-Y. Wei T.-T.. (2021). Neuraminidase 1 is a driver of experimental cardiac hypertrophy. Eur. Heart J. 42, 3770–3782. doi: 10.1093/eurheartj/ehab347 34179969

[B32] Cohen M. Varki A. (2010). The sialome—Far more than the sum of its parts. OMICS: A J. Integr. Biol. 14, 455–464. doi: 10.1089/omi.2009.0148 20726801

[B33] Cortés-Sarabia K. Rodríguez-Nava C. Medina-Flores Y. Mata-Ruíz O. López-Meza J. E. Gómez-Cervantes M. D.. (2020). Production and characterization of a monoclonal antibody against the sialidase of Gardnerella vaginalis using a synthetic peptide in a MAP8 format. Appl. Microbiol. Biotechnol. 104, 6173–6183. doi: 10.1007/s00253-020-10691-z 32462244 PMC7253150

[B34] Delgado-Diaz D. J. Tyssen D. Hayward J. A. Gugasyan R. Hearps A. C. Tachedjian G. (2020). Distinct immune responses elicited from cervicovaginal epithelial cells by lactic acid and short chain fatty acids associated with optimal and non-optimal vaginal microbiota. Front. Cell. Infect. Microbiol. 9. doi: 10.3389/fcimb.2019.00446 PMC6965070 31998660

[B35] Del Pozo J. L. (2018). Biofilm-related disease. Expert Rev. Anti-infective Ther. 16, 51–65. doi: 10.1080/14787210.2018.1417036 29235402

[B36] Di Paola M. Sani C. Clemente A. M. Iossa A. Perissi E. Castronovo G.. (2017). Characterization of cervico-vaginal microbiota in women developing persistent high-risk Human Papillomavirus infection. Sci. Rep. 7, 10200. doi: 10.1038/s41598-017-09842-6 28860468 PMC5579045

[B37] Doerflinger S. Y. Throop A. L. Herbst-Kralovetz M. M. (2014). Bacteria in the vaginal microbiome alter the innate immune response and barrier properties of the human vaginal epithelia in a species-specific manner. J. Infect. Dis. 209, 1989–1999. doi: 10.1093/infdis/jiu004 24403560

[B38] Duan S. Paulson J. C. (2020). Siglecs as immune cell checkpoints in disease. Annu. Rev. Immunol. 38, 365–395. doi: 10.1146/annurev-immunol-102419-035900 31986070

[B39] Eade C. R. Diaz C. Wood M. P. Anastos K. Patterson B. K. Gupta P.. (2012). Identification and characterization of bacterial vaginosis-associated pathogens using a comprehensive cervical-vaginal epithelial coculture assay. PloS One 7, e50106. doi: 10.1371/journal.pone.0050106 23166828 PMC3499514

[B40] Egan M. O’Connell Motherway M. Ventura M. van Sinderen D. (2014). Metabolism of sialic acid by bifidobacterium breve UCC2003. Appl. Environ. Microbiol. 80, 4414–4426. doi: 10.1128/AEM.01114-14 24814790 PMC4068672

[B41] Ferreira C. S. T. Marconi C. Parada C. M. G. L. Ravel J. Silva M. G. D. (2022). Sialidase activity in the cervicovaginal fluid is associated with changes in bacterial components of lactobacillus-deprived microbiota. Front. Cell. Infect. Microbiol. 11. doi: 10.3389/fcimb.2021.813520 PMC8793624 35096658

[B42] Flemming H.-C. Wingender J. Szewzyk U. Steinberg P. Rice S. A. Kjelleberg S. (2016). Biofilms: an emergent form of bacterial life. Nat. Rev. Microbiol. 14, 563–575. doi: 10.1038/nrmicro.2016.94 27510863

[B43] Foessleitner P. Kiss H. Deinsberger J. Ott J. Zierhut L. Rosta K.. (2021). Screening pregnant women for bacterial vaginosis using a point-of-care test: A prospective validation study. JCM 10, 2275. doi: 10.3390/jcm10112275 34073935 PMC8197407

[B44] Ghosh S. (2020). Sialic acid and biology of life: An introduction, in Sialic acids and sialoglycoconjugates in the biology of life, health and disease (Amsterdam: Elsevier), 1–61. doi: 10.1016/B978-0-12-816126-5.00001-9

[B45] Gilbert N. M. Lewis W. G. Lewis A. L. (2013). Clinical features of bacterial vaginosis in a murine model of vaginal infection with gardnerella vaginalis. PloS One 8, e59539. doi: 10.1371/journal.pone.0059539 23527214 PMC3602284

[B46] Gilbert N. M. Lewis W. G. Li G. Sojka D. K. Lubin J. B. Lewis A. L. (2019). Gardnerella vaginalis and Prevotella bivia Trigger Distinct and Overlapping Phenotypes in a Mouse Model of Bacterial Vaginosis. J. Infect. Dis. 220, 1099–1108. doi: 10.1093/infdis/jiy704 30715405 PMC6736442

[B47] Glanz V. Y. Myasoedova V. A. Grechko A. V. Orekhov A. N. (2018). Inhibition of sialidase activity as a therapeutic approach. DDDT Volume 12, 3431–3437. doi: 10.2147/DDDT.S176220 PMC6186905 30349196

[B48] Govinden G. Parker J. L. Naylor K. L. Frey A. M. Anumba D. O. C. Stafford G. P. (2018). Inhibition of sialidase activity and cellular invasion by the bacterial vaginosis pathogen Gardnerella vaginalis. Arch. Microbiol. 200, 1129–1133. doi: 10.1007/s00203-018-1520-4 29777255 PMC6096708

[B49] Haines-Menges B. L. Whitaker W. B. Lubin J. B. Boyd E. F. (2015). Host sialic acids: A delicacy for the pathogen with discerning taste. Microbiol. Spectr. 3, 3.4.07. doi: 10.1128/microbiolspec.MBP-0005-2014 PMC6089508 26350327

[B50] Hardy L. Jespers V. Van Den Bulck M. Buyze J. Mwambarangwe L. Musengamana V.. (2017). The presence of the putative Gardnerella vaginalis sialidase A gene in vaginal specimens is associated with bacterial vaginosis biofilm. PloS One 12, e0172522. doi: 10.1371/journal.pone.0172522 28241058 PMC5328246

[B51] Hayden F. G. Treanor J. J. Fritz R. S. Lobo M. Betts R. F. Miller M.. (1999). Use of the oral neuraminidase inhibitor oseltamivir in experimental human influenza: randomized controlled trials for prevention and treatment. JAMA 282, 1240. doi: 10.1001/jama.282.13.1240 10517426

[B52] He Y. Na R. Niu X. Xiao B. Yang H. (2021). Lactobacillus rhamnosus and Lactobacillus casei Affect Various Stages of Gardnerella Species Biofilm Formation. Front. Cell. Infect. Microbiol. 11. doi: 10.3389/fcimb.2021.568178 PMC7933028 33680986

[B53] Honest H. Bachmann L. M. Knox E. M. Gupta J. K. Kleijnen J. Khan K. S. (2004). The accuracy of various tests for bacterial vaginosis in predicting preterm birth: a systematic review. BJOG 111, 409–422. doi: 10.1111/j.1471-0528.2004.00124.x 15104603

[B54] Howe L. Wiggins R. Soothill P. W. Millar M. R. Horner P. J. Corfield A. P. (1999). Mucinase and sialidase activity of the vaginal microflora: implications for the pathogenesis of preterm labour. Int. J. STD AIDS 10, 442–447. doi: 10.1258/0956462991914438 10454178

[B55] Huang Y.-L. Chassard C. Hausmann M. Von Itzstein M. Hennet T. (2015). Sialic acid catabolism drives intestinal inflammation and microbial dysbiosis in mice. Nat. Commun. 6, 8141. doi: 10.1038/ncomms9141 26303108 PMC4560832

[B56] Janulaitiene M. Gegzna V. Baranauskiene L. Bulavaitė A. Simanavicius M. Pleckaityte M. (2018). Phenotypic characterization of Gardnerella vaginalis subgroups suggests differences in their virulence potential. PloS One 13, e0200625. doi: 10.1371/journal.pone.0200625 30001418 PMC6042761

[B57] Javanian M. Barary M. Ghebrehewet S. Koppolu V. Vasigala V. Ebrahimpour S. (2021). A brief review of influenza virus infection. J. Med. Virol. 93, 4638–4646. doi: 10.1002/jmv.26990 33792930

[B58] Javed A. Parvaiz F. Manzoor S. (2019). Bacterial vaginosis: An insight into the prevalence, alternative treatments regimen and it’s associated resistance patterns. Microbial Pathogenesis 127, 21–30. doi: 10.1016/j.micpath.2018.11.046 30502515

[B59] Joo H.-S. Otto M. (2012). Molecular basis of *in vivo* biofilm formation by bacterial pathogens. Chem. Biol. 19, 1503–1513. doi: 10.1016/j.chembiol.2012.10.022 23261595 PMC3530155

[B60] Jung H.-S. Ehlers M. M. Lombaard H. Redelinghuys M. J. Kock M. M. (2017). Etiology of bacterial vaginosis and polymicrobial biofilm formation. Crit. Rev. Microbiol. 43, 651–667. doi: 10.1080/1040841X.2017.1291579 28358585

[B61] Karhadkar T. R. Chen W. Pilling D. Gomer R. H. (2022). Inhibitors of the sialidase NEU3 as potential therapeutics for fibrosis. IJMS 24, 239. doi: 10.3390/ijms24010239 36613682 PMC9820515

[B62] Keil J. M. Rafn G. R. Turan I. M. Aljohani M. A. Sahebjam-Atabaki R. Sun X.-L. (2022). Sialidase inhibitors with different mechanisms. J. Med. Chem. 65, 13574–13593. doi: 10.1021/acs.jmedchem.2c01258 36252951 PMC9620260

[B63] Ketterer M. R. Rice P. A. Gulati S. Kiel S. Byerly L. Fortenberry J. D.. (2016). Desialylation of *neisseria gonorrhoeae* lipooligosaccharide by cervicovaginal microbiome sialidases: the potential for enhancing infectivity in men. J. Infect. Dis. 214, 1621–1628. doi: 10.1093/infdis/jiw329 27471322 PMC6392504

[B64] Khan A. Das S. Sergi C. (2021). Therapeutic potential of neu1 in alzheimer’s disease *via* the immune system. Am. J. Alzheimers Dis. Other Demen 36, 153331752199614. doi: 10.1177/1533317521996147 PMC10624071 33719595

[B65] Kim B. R. Park J.-Y. Jeong H. J. Kwon H.-J. Park S.-J. Lee I.-C.. (2018). Design, synthesis, and evaluation of curcumin analogues as potential inhibitors of bacterial sialidase. J. Enzyme Inhibition Medicinal Chem. 33, 1256–1265. doi: 10.1080/14756366.2018.1488695 PMC6104608 30126306

[B66] Kurukulasuriya S. P. Patterson M. H. Hill J. E. (2021). Slipped-strand mispairing in the gene encoding sialidase nanH3 in gardnerella spp. Infect. Immun. 89, e00583–e00520. doi: 10.1128/IAI.00583-20 33361200 PMC8097274

[B67] Lewis A. L. Lewis W. G. (2012). Host sialoglycans and bacterial sialidases: a mucosal perspective. Cell. Microbiol. 14, 1174–1182. doi: 10.1111/j.1462-5822.2012.01807.x 22519819

[B68] Lewis W. G. Robinson L. S. Gilbert N. M. Perry J. C. Lewis A. L. (2013). Degradation, foraging, and depletion of mucus sialoglycans by the vagina-adapted actinobacterium gardnerella vaginalis. J. Biol. Chem. 288, 12067–12079. doi: 10.1074/jbc.M113.453654 23479734 PMC3636892

[B69] Lewis W. G. Robinson L. S. Perry J. Bick J. L. Peipert J. F. Allsworth J. E.. (2012). Hydrolysis of secreted sialoglycoprotein immunoglobulin A (IgA) in ex vivo and biochemical models of bacterial vaginosis. J. Biol. Chem. 287, 2079–2089. doi: 10.1074/jbc.M111.278135 22134918 PMC3265887

[B70] Liao H. Klaus C. Neumann H. (2020). Control of innate immunity by sialic acids in the nervous tissue. IJMS 21, 5494. doi: 10.3390/ijms21155494 32752058 PMC7432451

[B71] Libby E. K. Pascal K. E. Mordechai E. Adelson M. E. Trama J. P. (2008). Atopobium vaginae triggers an innate immune response in an in *vitro* model of bacterial vaginosis. Microbes Infection 10, 439–446. doi: 10.1016/j.micinf.2008.01.004 18403235

[B72] Liu G. Wang B. Zhang Y. Xing G. Yang X. Wang S. (2018). A tetravalent sialic acid-coated tetraphenylethene luminogen with aggregation-induced emission characteristics: design, synthesis and application for sialidase activity assay, high-throughput screening of sialidase inhibitors and diagnosis of bacterial vaginosis. Chem. Commun. 54, 10691–10694. doi: 10.1039/C8CC06300A 30187046

[B73] Liu X. Zhang Y. Yu W. Zhang W. Jiang J. Gu Q.. (2023). Evaluating the activity of neuraminidase in bacterial vaginosis microflora and imaging sialic acid on the cell membrane by boron and nitrogen codoped fluorescent carbon dots. ACS Sens. 8, 2556–2562. doi: 10.1021/acssensors.3c00219 37322599

[B74] Lo A. W. Seers C. A. Boyce J. D. Dashper S. G. Slakeski N. Lissel J. P.. (2009). Comparative transcriptomic analysis of Porphyromonas gingivalisbiofilm and planktonic cells. BMC Microbiol. 9, 18. doi: 10.1186/1471-2180-9-18 19175941 PMC2637884

[B75] Lopes Dos Santos Santiago G. Deschaght P. El Aila N. Kiama T. N. Verstraelen H. Jefferson K. K.. (2011). Gardnerella vaginalis comprises three distinct genotypes of which only two produce sialidase. Am. J. Obstet. Gynecol. 204, 450.e1–450.e7. doi: 10.1016/j.ajog.2010.12.061 21444061

[B76] Ma X. Wang X. Ye S. Liu J. Yuan H. Wang N. (2022). Biofilm and pathogenic factor analysis of Gardnerella vaginalis associated with bacterial vaginosis in Northeast China. Front. Microbiol. 13. doi: 10.3389/fmicb.2022.1033040 PMC9815022 36619994

[B77] Mabugana M. C. Dias B. D. C. Muller E. E. Kufa T. Gumede L. Mahlangu M. P.. (2023). The evaluation of the Allplex^TM^ BV molecular assay for the diagnosis of bacterial vaginosis in symptomatic South African females. Diagn. Microbiol. Infect. Dis. 106, 115924. doi: 10.1016/j.diagmicrobio.2023.115924 37030281

[B78] Madhivanan P. Krupp K. Li T. Ravi K. Selezneva J. Srinivas V.. (2014). Performance of BVBlue rapid test in detecting bacterial vaginosis among women in mysore, India. Infect. Dis. Obstet. Gynecol. 2014, 1–7. doi: 10.1155/2014/908313 PMC3913452 24526829

[B79] Manns-James L. (2011). Bacterial vaginosis and preterm birth. J. Midwife Womens Health 56, 575–583. doi: 10.1111/j.1542-2011.2011.00086.x 22060217

[B80] Moncla B. J. Chappell C. A. Debo B. M. Meyn L. A. (2016). The effects of hormones and vaginal microflora on the glycome of the female genital tract: cervical-vaginal fluid. PloS One 11, e0158687. doi: 10.1371/journal.pone.0158687 27437931 PMC4954690

[B81] Moncla B. J. Chappell C. A. Mahal L. K. Debo B. M. Meyn L. A. Hillier S. L. (2015). Impact of bacterial vaginosis, as assessed by nugent criteria and hormonal status on glycosidases and lectin binding in cervicovaginal lavage samples. PloS One 10, e0127091. doi: 10.1371/journal.pone.0127091 26011704 PMC4444347

[B82] Moran A. P. Gupta A. Joshi L. (2011). Sweet-talk: role of host glycosylation in bacterial pathogenesis of the gastrointestinal tract. Gut 60, 1412–1425. doi: 10.1136/gut.2010.212704 21228430

[B83] Morrill S. R. Saha S. Varki A. P. Lewis W. G. Ram S. Lewis A. L. (2023). Gardnerella vaginolysin potentiates glycan molecular mimicry by neisseria gonorrhoeae. J. Infect. Dis. 228, 1610–1620. doi: 10.1093/infdis/jiad391 37722688 PMC10681867

[B84] Myziuk L. Romanowski B. Johnson S. C. (2003). BVBlue test for diagnosis of bacterial vaginosis. J. Clin. Microbiol. 41, 1925–1928. doi: 10.1128/JCM.41.5.1925-1928.2003 12734228 PMC154737

[B85] Natori Y. Ohkura N. Nasui M. Atsumi G. Kihara-Negishi F. (2013). Acidic sialidase activity is aberrant in obese and diabetic mice. Biol. Pharm. Bull. 36, 1027–1031. doi: 10.1248/bpb.b12-00995 23727924

[B86] Ng S. Chen M. Kundu S. Wang X. Zhou Z. Zheng Z.. (2021). Large-scale characterisation of the pregnancy vaginal microbiome and sialidase activity in a low-risk Chinese population. NPJ Biofilms Microbiomes 7, 89. doi: 10.1038/s41522-021-00261-0 34930922 PMC8688454

[B87] Novak J. Belleti R. da Silva Pinto G. V. do Nascimento Bolpetti A. da Silva M. G. Marconi C. (2023). Cervicovaginal Gardnerella sialidase-encoding gene in persistent human papillomavirus infection. Sci. Rep. 13, 14266. doi: 10.1038/s41598-023-41469-8 37652960 PMC10471596

[B88] Nugent R. P. Krohn M. A. Hillier S. L. (1991). Reliability of diagnosing bacterial vaginosis is improved by a standardized method of gram stain interpretation. J. Clin. Microbiol. 29, 297–301. doi: 10.1128/jcm.29.2.297-301.1991 1706728 PMC269757

[B89] Ochs M. Hegermann J. Lopez-Rodriguez E. Timm S. Nouailles G. Matuszak J.. (2020). On top of the alveolar epithelium: surfactant and the glycocalyx. IJMS 21, 3075. doi: 10.3390/ijms21093075 32349261 PMC7246550

[B90] Permsak S. Chananan K. Suthee P. (2005). BVBLUE test for diagnosis of bacterial vaginosis in pregnant women attending antenatal care at Phramongkutklao Hospital. J. Med. Assoc. Thai. 88 Suppl 3, S7–13. 16858939

[B91] Pezzicoli A. Ruggiero P. Amerighi F. Telford J. L. Soriani M. (2012). Exogenous sialic acid transport contributes to group B streptococcus infection of mucosal surfaces. J. Infect. Dis. 206, 924–931. doi: 10.1093/infdis/jis451 22829646

[B92] Poole J. Day C. J. Von Itzstein M. Paton J. C. Jennings M. P. (2018). Glycointeractions in bacterial pathogenesis. Nat. Rev. Microbiol. 16, 440–452. doi: 10.1038/s41579-018-0007-2 29674747

[B93] Pshezhetsky A. V. Ashmarina L. I. (2013). Desialylation of surface receptors as a new dimension in cell signaling. Biochem. Moscow 78, 736–745. doi: 10.1134/S0006297913070067 24010837

[B94] Qin H. Xiao B. (2022). Research progress on the correlation between gardnerella typing and bacterial vaginosis. Front. Cell. Infect. Microbiol. 12. doi: 10.3389/fcimb.2022.858155 PMC8990036 35402309

[B95] Ram S. Shaughnessy J. De Oliveira R. B. Lewis L. A. Gulati S. Rice P. A. (2017). Gonococcal lipooligosaccharide sialylation: virulence factor and target for novel immunotherapeutics. Pathog. Dis. 75, ftx049. doi: 10.1093/femspd/ftx049 28460033 PMC5449626

[B96] Ravel J. Gajer P. Abdo Z. Schneider G. M. Koenig S. S. K. McCulle S. L.. (2011). Vaginal microbiome of reproductive-age women. Proc. Natl. Acad. Sci. U. S. A. 108, 4680–4687. doi: 10.1073/pnas.1002611107 20534435 PMC3063603

[B97] Ravel J. Moreno I. Simón C. (2021). Bacterial vaginosis and its association with infertility, endometritis, and pelvic inflammatory disease. Am. J. Obstet. Gynecol. 224, 251–257. doi: 10.1016/j.ajog.2020.10.019 33091407

[B98] Robinson L. S. Schwebke J. Lewis W. G. Lewis A. L. (2019). Identification and characterization of NanH2 and NanH3, enzymes responsible for sialidase activity in the vaginal bacterium Gardnerella vaginalis. J. Biol. Chem. 294, 5230–5245. doi: 10.1074/jbc.RA118.006221 30723162 PMC6462536

[B99] Rodríguez-Nava C. Cortés-Sarabia K. Avila-Huerta M. D. Ortiz-Riaño E. J. Estrada-Moreno A. K. Alarcón-Romero L. D. C.. (2021). Nanophotonic sialidase immunoassay for bacterial vaginosis diagnosis. ACS Pharmacol. Transl. Sci. 4, 365–371. doi: 10.1021/acsptsci.0c00211 33615186 PMC7887842

[B100] Roggentin P. Rothe B. Kaper J. B. Galen J. Lawrisuk L. Vimr E. R.. (1989). Conserved sequences in bacterial and viral sialidases. Glycoconjugate J. 6, 349–353. doi: 10.1007/BF01047853 2562507

[B101] Santos C. M. A. Pires M. C. V. Leão T. L. Silva A. K. S. Miranda L. S. Martins F. S.. (2018). Anti-inflammatory effect of two Lactobacillus strains during infection with Gardnerella vaginalis and Candida albicans in a HeLa cell culture model. Microbiology 164, 349–358. doi: 10.1099/mic.0.000608 29458690

[B102] Schauer R. Kamerling J. P. (1997). Chemistry, biochemistry and biology of sialic acids, in New comprehensive biochemistry (Amsterdam: Elsevier), 243–402. doi: 10.1016/S0167-7306(08)60624-9

[B103] Schauer R. Kamerling J. P. (2018). Exploration of the sialic acid world, in Advances in carbohydrate chemistry and biochemistry (Amsterdam: Elsevier), 1–213. doi: 10.1016/bs.accb.2018.09.001 PMC7112061 30509400

[B104] Schellenberg J. J. Paramel Jayaprakash T. Withana Gamage N. Patterson M. H. Vaneechoutte M. Hill J. E. (2016). Gardnerella vaginalis Subgroups Defined by cpn60 Sequencing and Sialidase Activity in Isolates from Canada, Belgium and Kenya. PloS One 11, e0146510. doi: 10.1371/journal.pone.0146510 26751374 PMC4709144

[B105] Schwebke J. R. Muzny C. A. Josey W. E. (2014). Role of gardnerella vaginalis in the pathogenesis of bacterial vaginosis: A conceptual model. J. Infect. Dis. 210, 338–343. doi: 10.1093/infdis/jiu089 24511102

[B106] Shipitsyna E. Krysanova A. Khayrullina G. Shalepo K. Savicheva A. Guschin A.. (2019). Quantitation of all Four Gardnerella vaginalis Clades Detects Abnormal Vaginal Microbiota Characteristic of Bacterial Vaginosis More Accurately than Putative G. vaginalis Sialidase A Gene Count. Mol. Diagn. Ther. 23, 139–147. doi: 10.1007/s40291-019-00382-5 30721449 PMC6394432

[B107] Siegel S. J. Roche A. M. Weiser J. N. (2014). Influenza promotes pneumococcal growth during coinfection by providing host sialylated substrates as a nutrient source. Cell Host Microbe 16, 55–67. doi: 10.1016/j.chom.2014.06.005 25011108 PMC4096718

[B108] Slomiany B. L. Murty V. L. N. Piotrowski J. Slomiany A. (1996). Salivary mucins in oral mucosal defense. Gen. Pharmacology: Vasc. System 27, 761–771. doi: 10.1016/0306-3623(95)02050-0 8842677

[B109] Smayevsky J. Canigia L. F. Lanza A. Bianchini H. (2001). Vaginal microflora associated with bacterial vaginosis in nonpregnant women: reliability of sialidase detection. Infect. Dis. Obstet. Gynecol. 9, 17–22. doi: 10.1155/S1064744901000047 11368254 PMC1784631

[B110] SoÈnmez H. SuÈer S. GuÈngoÈr Z. BalogÏlu H. KoÈkogÏlu E. (1999). Tissue and serum sialidase levels in breast cancer. Cancer Lett. 136, 75–78. doi: 10.1016/S0304-3835(98)00295-X 10211942

[B111] Soong G. (2006). Bacterial neuraminidase facilitates mucosal infection by participating in biofilm production. J. Clin. Invest. 116, 2297–2305. doi: 10.1172/JCI27920 16862214 PMC1513050

[B112] Srinivasan S. Morgan M. T. Fiedler T. L. Djukovic D. Hoffman N. G. Raftery D.. (2015). Metabolic signatures of bacterial vaginosis. mBio 6, e00204–e00215. doi: 10.1128/mBio.00204-15 25873373 PMC4453549

[B113] Swidsinski A. Mendling W. Loening-Baucke V. Ladhoff A. Swidsinski S. Hale L. P.. (2005). Adherent biofilms in bacterial vaginosis. Obstet. Gynecol. 106, 1013–1023. doi: 10.1097/01.AOG.0000183594.45524.d2 16260520

[B114] Swidsinski A. Mendling W. Loening-Baucke V. Swidsinski S. Dörffel Y. Scholze J.. (2008). An adherent Gardnerella vaginalis biofilm persists on the vaginal epithelium after standard therapy with oral metronidazole. Am. J. Obstet. Gynecol. 198, 97.e1–97.e6. doi: 10.1016/j.ajog.2007.06.039 18005928

[B115] Taylor B. D. Darville T. Haggerty C. L. (2013). Does bacterial vaginosis cause pelvic inflammatory disease? Sexually Transmitted Dis. 40, 117–122. doi: 10.1097/OLQ.0b013e31827c5a5b 23324974

[B116] Varki A. (2009). Multiple changes in sialic acid biology during human evolution. Glycoconj J. 26, 231–245. doi: 10.1007/s10719-008-9183-z 18777136 PMC7087641

[B117] Varki A. Gagneux P. (2012). Multifarious roles of sialic acids in immunity. Ann. New York Acad. Sci. 1253, 16–36. doi: 10.1111/j.1749-6632.2012.06517.x 22524423 PMC3357316

[B118] Verstraelen H. Swidsinski A. (2013). The biofilm in bacterial vaginosis: implications for epidemiology, diagnosis and treatment. Curr. Opin. Infect. Dis. 26, 86. doi: 10.1097/QCO.0b013e32835c20cd 23221767

[B119] Vimr E. Lichtensteiger C. (2002). To sialylate, or not to sialylate: that is the question. Trends Microbiol. 10, 254–257. doi: 10.1016/S0966-842X(02)02361-2 12088651

[B120] Vitali B. Cruciani F. Picone G. Parolin C. Donders G. Laghi L. (2015). Vaginal microbiome and metabolome highlight specific signatures of bacterial vaginosis. Eur. J. Clin. Microbiol. Infect. Dis. 34, 2367–2376. doi: 10.1007/s10096-015-2490-y 26385347

[B121] Wang L. Koppolu S. Chappell C. Moncla B. J. Hillier S. L. Mahal L. K. (2015). Studying the effects of reproductive hormones and bacterial vaginosis on the glycome of lavage samples from the cervicovaginal cavity. PloS One 10, e0127021. doi: 10.1371/journal.pone.0127021 25993513 PMC4439148

[B122] Wiggins R. (2001). Mucinases and sialidases: their role in the pathogenesis of sexually transmitted infections in the female genital tract. Sex. Transm. Infect. 77, 402–408. doi: 10.1136/sti.77.6.402 11714935 PMC1744407

[B123] Wong A. Grau M. A. Singh A. K. Woodiga S. A. King S. J. (2018). Role of neuraminidase-producing bacteria in exposing cryptic carbohydrate receptors for streptococcus gordonii adherence. Infect. Immun. 86, e00068–e00018. doi: 10.1128/IAI.00068-18 29661931 PMC6013669

[B124] Woo H. S. Kim D. W. Curtis-Long M. J. Lee B. W. Lee J. H. Kim J. Y.. (2011). Potent inhibition of bacterial neuraminidase activity by pterocarpans isolated from the roots of Lespedeza bicolor. Bioorganic Medicinal Chem. Lett. 21, 6100–6103. doi: 10.1016/j.bmcl.2011.08.046 21911291

[B125] Wu S. Lin X. Hui K. M. Yang S. Wu X. Tan Y.. (2019). A biochemiluminescent sialidase assay for diagnosis of bacterial vaginosis. Sci. Rep. 9, 20024. doi: 10.1038/s41598-019-56371-5 31882933 PMC6934538

[B126] Xu X. Tong T. Yang X. Pan Y. Lin L. Li C. (2017). Differences in survival, virulence and biofilm formation between sialidase-deficient and W83 wild-type Porphyromonas gingivalis strains under stressful environmental conditions. BMC Microbiol. 17, 178. doi: 10.1186/s12866-017-1087-2 28821225 PMC5563019

[B127] Yoo E. M. Morrison S. L. (2005). IgA: An immune glycoprotein. Clin. Immunol. 116, 3–10. doi: 10.1016/j.clim.2005.03.010 15925826

[B128] Zambon M. C. (2001). The pathogenesis of influenza in humans. Rev. Med. Virol. 11, 227–241. doi: 10.1002/rmv.319 11479929

[B129] Zhang Y. Rochefort D. (2013). Fast and effective paper based sensor for self-diagnosis of bacterial vaginosis. Analytica Chimica Acta 800, 87–94. doi: 10.1016/j.aca.2013.09.032 24120172

[B130] Zhang L. Wei T.-T. Li Y. Li J. Fan Y. Huang F.-Q.. (2018). Functional metabolomics characterizes a key role for *N* -acetylneuraminic acid in coronary artery diseases. Circulation 137, 1374–1390. doi: 10.1161/CIRCULATIONAHA.117.031139 29212895

[B131] Zhou X. Zhai Y. Liu C. Yang G. Guo J. Li G.. (2020). Sialidase NEU1 suppresses progression of human bladder cancer cells by inhibiting fibronectin-integrin α5β1 interaction and Akt signaling pathway. Cell Commun. Signal 18, 44. doi: 10.1186/s12964-019-0500-x 32164705 PMC7066847

